# Managing insecticide resistance in malaria vectors by combining carbamate-treated plastic wall sheeting and pyrethroid-treated bed nets

**DOI:** 10.1186/1475-2875-8-233

**Published:** 2009-10-20

**Authors:** Armel Djènontin, Joseph Chabi, Thierry Baldet, Seth Irish, Cédric Pennetier, Jean-Marc Hougard, Vincent Corbel, Martin Akogbéto, Fabrice Chandre

**Affiliations:** 1 CREC, Cotonou, Bénin; 2 CREC/IRD UR016, Cotonou, Bénin; 3 LIN/IRD UR016, Montpellier, France; 4 London School of Tropical Medicine and Hygiene, London, UK

## Abstract

**Background:**

Pyrethroid resistance is now widespread in *Anopheles gambiae*, the major vector for malaria in sub-Saharan Africa. This resistance may compromise malaria vector control strategies that are currently in use in endemic areas. In this context, a new tool for management of resistant mosquitoes based on the combination of a pyrethroid-treated bed net and carbamate-treated plastic sheeting was developed.

**Methods:**

In the laboratory, the insecticidal activity and wash resistance of four carbamate-treated materials: a cotton/polyester blend, a polyvinyl chloride tarpaulin, a cotton/polyester blend covered on one side with polyurethane, and a mesh of polypropylene fibres was tested. These materials were treated with bendiocarb at 100 mg/m^2 ^and 200 mg/m^2 ^with and without a binding resin to find the best combination for field studies. Secondly, experimental hut trials were performed in southern Benin to test the efficacy of the combined use of a pyrethroid-treated bed net and the carbamate-treated material that was the most wash-resistant against wild populations of pyrethroid-resistant *An. gambiae *and *Culex quinquefasciatus*.

**Results:**

Material made of polypropylene mesh (PPW) provided the best wash resistance (up to 10 washes), regardless of the insecticide dose, the type of washing, or the presence or absence of the binding resin. The experimental hut trial showed that the combination of carbamate-treated PPW and a pyrethroid-treated bed net was extremely effective in terms of mortality and inhibition of blood feeding of pyrethroid-resistant *An. gambiae*. This efficacy was found to be proportional to the total surface of the walls. This combination showed a moderate effect against wild populations of *Cx. quinquefasciatus*, which were strongly resistant to pyrethroid.

**Conclusion:**

These preliminary results should be confirmed, including evaluation of entomological, parasitological, and clinical parameters. Selective pressure on resistance mechanisms within the vector population, effects on other pest insects, and the acceptability of this management strategy in the community also need to be evaluated.

## Background

Malaria is one of the main public health problems in Africa, causing more than one million deaths per year and placing a strong burden on developing African countries [1]. Vector control remains an important component of malaria prevention. The two main methods of malarial vector control are indoor residual spraying (IRS) and insecticide-treated nets (ITNs). The choice of method depends not only on the epidemiological setting and the strategic objectives of vector control, but also on the feasibility and existence of an appropriate delivery structure. In most countries of sub-Saharan Africa, where malaria transmission is stable and infrastructures for large-scale IRS do not exist, ITNs are more cost-effective. Recently, the development of long-lasting insecticidal nets (LLINs), which resist loss of insecticide during washing and extend the residual efficacy of the insecticide, has addressed the technical and logistical constraints associated with re-impregnation of insecticide on the nets. During the last decade, LLINs have become the predominant method of preventing malaria in many malaria-affected countries [1,2]. More than eighty studies carried out around the world have shown the effectiveness of treated nets in reducing the incidence of malaria morbidity by 50% [2]. In Benin, the National Malaria Control Programme of the Ministry of Health has implemented a large campaign of LLIN distribution to pregnant women and children younger than five years of age, the groups most affected by malaria [1].

Pyrethroids are the only insecticides used for the treatment of nets because of their high efficacy and fast effect at low doses, their excito-repellent properties, their residual effects, and their low toxicity in mammals [3]. Unfortunately, resistance to pyrethroids is now widespread among *Anopheles gambiae*, the main malarial vector, notably in West African countries, including Cote d'Ivoire [4], Burkina Faso [5,6], Ghana [7], Nigeria [8], Mali [9], and Benin [10,11]. This resistance is due to a target site modification [12] and/or an increase in the ability of the mosquitoes to metabolize the insecticide (metabolic resistance). Target site modification is based on point mutations in a voltage-gated sodium channel (L1014F in West Africa and L1014S in East Africa) that confers cross-resistance to pyrethroids and DDT [13,14] Pyrethroid resistance based on metabolic detoxification is mainly due to an increase in the activity of mono-oxygenases and secondarily to an increase of the activity of esterases [11,15].

Many questions remain about the impact of pyrethroid resistance on the effectiveness of LLINs against malarial vectors. To this point, studies performed in areas with pyrethroid-resistant mosquitoes (e.g., Cote d'Ivoire) have not shown a significant decrease in the effectiveness of pyrethroid-treated mosquito nets, either entomologically [16,17] or parasitologically and clinically [18]. However, a recent study in experimental huts in a *kdr*-type-resistant area in the southern part of Benin (Ladji) showed dramatic decreases in ITN and IRS efficacies against *An. gambiae *in terms of mortality rate and prevention of bites as compared to a northern area without resistance (Malanville) [19]. These results show that it is urgent to develop alternative strategies of vector control to maintain efficacy against resistant mosquitoes and also to limit the expansion of pyrethroid resistance in malaria vectors.

In this context, the efficacy of a new concept of malaria vector control by combining carbamate-treated wall plastic sheeting and pyrethroid-treated nets (ITNs) was studied. Mixing insecticides with different modes of action is one resistance management strategy. In contrast to previous "two-in-one" strategies, which combine two insecticides with different modes of action onto the same mosquito net [20-22], this concept has the advantage of reducing human contact with carbamates, a class of insecticides that is not recommended by the WHO for bed net impregnation. It combines the blood feeding inhibition effect of ITNs and the lethal effect of carbamate-treated plastic sheeting, which mimics a long lasting indoor residual spraying.

In the present study, first, the efficacy and wash resistance of different materials treated with bendiocarb (a carbamate approved by the WHO for IRS) at the doses of 100 mg/m^2 ^and 200 mg/m^2^, with or without a binding resin, were evaluated in the laboratory. Then, the material that had the best wash resistance, as determined by laboratory trials was tested in experimental huts in combination with deltamethrin-treated nets against *kdr*-resistant populations of *An. gambiae *and *Culex quinquefasciatus*.

## Methods

### Laboratory evaluation

#### Mosquitoes

The pyrethroid-susceptible "Kisumu" strain of *An. gambiae*, originated from Kenya, was used for the bioassays. This reference strain is maintained at the insectary of the Entomological Research Centre of Cotonou (CREC).

#### Insecticides

A wettable powder (WP 80 W FICAM) formulation of bendiocarb was used for the treatment of netting materials. This insecticide is an irreversible acetylcholinesterase inhibitor acting on the insect central nervous system [23]. A fixing resin was used to ease the impregnation of the material with a brush and to improve its wash resistance. Bendiocarb and the fixing resin were provided by Bayer Environmental Science (Lyon, France).

#### Treatment and washing of the materials

The following materials were used for the study:

1) blue fabric made of 35% cotton and 65% polyester (CP);

2) tent material made of the same fabric, but covered on one side with waterproof polyurethane (CPPU);

3) rice sack from the local market, made of woven polypropylene (fibres 2 mm wide; PPW);

4) thick waterproof tarpaulin made of polyvinyl chloride (PVC).

Each type of material was cut into 25 cm × 25 cm pieces and then treated with bendiocarb at 100 mg/m^2 ^or 200 mg/m^2 ^using a brush. These doses have been recommended by the WHO for classical IRS [24]. The doses tested were lower than the 400 mg/m^2 ^recommended for IRS and are based on a Human Risk Assessment established by toxicologists from Bayer Crop Science considering that there was no unacceptable risk at these concentrations after accidental manipulation of treated plastic sheeting without any precaution by people in treated houses. Half of the pieces of material also received a binding resin at a dose of 12 ml/m^2^.

The washes were performed horizontally by hand using a sponge soaked in either water alone or water and "Savon de Marseille" soap (2 g/L). The pH of the soapy water was 9.7 and the water alone was 6.0. For each wash, the wet sponge was rubbed against all parts of the material three times over the course of about 10 seconds. For the washes using soapy water, another sponge with water alone was used to rinse the material. This sponge was passed over the entire piece of material once for three seconds. The material was dried horizontally on a table in the laboratory and left at ambient temperature for 24 hours. After drying, the treated materials were wrapped and kept in the refrigerator (4°C) until use in bioassays.

#### Bioassays

The efficacy of each treatment was evaluated using WHO cone tests [25]. This test consists of introducing unfed five-day-old mosquitoes into a Plexiglas cone attached to the insecticide-treated material. Fifteen mosquitoes were placed in each cone, and four cones were used for each type of material (n = 60). The contact time was 30 minutes. After exposure, the mosquitoes were placed in small cups, provided with sugar solution and maintained at 27 ± 2°C with a relative humidity of 80 ± 10% for 24 hours to assess delayed mortality. Actually, the real concentration of insecticide on treated material after each wash is not known.

### Experimental hut evaluation

#### Study area and huts design

**Figure 1:**
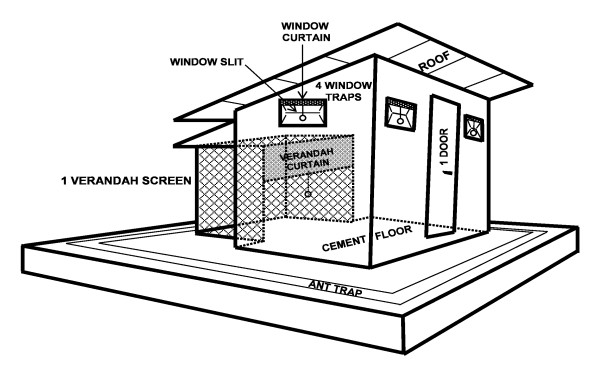
**Design of the experimental huts used in West Africa**.

This study was performed in experimental huts according to WHO protocol [25], in southern Benin, in a village situated near Lake Nokoué, about 30 kilometres from Cotonou. Four experimental huts, similar to typical African houses, were used [25,26]. They were 2.5 m long and 1.75 m wide, and the ceiling was 2 m high (Figure 1) [27]. The huts were built using cement bricks, and the floors and walls were covered with thin layers of cement. A plastic tarpaulin ceiling was stretched beneath the roof to protect sleeping residents from the heat and to facilitate the capture of mosquitoes. Each hut was surrounded with a moat to prevent the entry of ants and spiders, which can eat or carry away mosquitoes. The only possible exit for mosquitoes that had entered the hut was a veranda trap, opposite the door. For the present study, the entrances of the huts were closed to prevent the entry of wild mosquitoes.

#### Insecticidal treatment in the huts

Previous studies have always demonstrated that carbamates (and organophosphates), even though used on bed net, conferred a very weak personal protection. Indeed, these non-irritant insecticides seem to facilitate mosquitoes to take a blood meal through the net, hence conferring less personal protection [28,29]. Then, considering that mosquitoes will be released in each hut per night, the low personal protection conferred by the carbamates (previously demonstrated), and for ethical reasons, no hut in this study was without net.

Each hut was randomly allocated one of four treatments:

1) a hut with an untreated mosquito net (control);

2) a hut with a mosquito net treated with deltamethrin at 25 mg/m^2 ^(WHO recommended dose);

3) a hut with the top third of the walls covered with the carbamate-treated material that gave the best wash resistance in combination with the mosquito net treated with deltamethrin at 25 mg/m^2^;

4) a hut with all of the walls covered with the carbamate-treated material that gave the best wash resistance in combination with the mosquito net treated with deltamethrin at 25 mg/m^2^.

Nets were made of 100-denier polyester with a mesh size of 156 threads per square inch. The nets were manually impregnated with deltamethrin. To simulate the conditions of bed net wear and tear that can be encountered in the field, 6 holes, 4 × 4 cm each, were cut on the sides and ends of each net.

#### Mosquito species

Two local populations of pyrethroid-resistant *An. gambiae *and *Cx. quinquefasciatus*, collected as larvae and raised to adulthood in the insectary were used. Both populations have strong resistance to pyrethroids due to the *kdr *mutation and likely metabolism mechanism [10].

#### Release-recapture

Four adult volunteers were recruited from the local population. After having announced throughout the district that this project required volunteers, four adults were selected with the approval of the traditional head of the district. Volunteers were informed of the objective of this study and signed informed consent (or authorized a witness if illiterate). Each night, 100 *An. gambiae *females or 150 *Cx. quinquefasciatus *females, aged 5 days and never having had a blood meal, were released in each hut at 20:00, fifteen minutes before the entry of the volunteers, to acclimate. The volunteers slept in the huts from 20:15 to 08:00 the following morning. At 08:00, all mosquitoes were removed from the huts and their locations (hut vs. veranda), and the physiological status (dead/living and fed/unfed) were recorded. The surviving mosquitoes were placed in small cups and provided with honey solution for 24 hours to assess delayed mortality. Three replicates were performed for each experimental hut and each species (with a total of 300 *An. gambiae *and 450 *Cx. quinquefasciatus *per treatment).

Even though the mosquitoes released in the huts were raised in the insectary (without infection), the volunteers were provided with free and rapid treatment, as recommended by the WHO, in the case of any symptoms of malaria. Ethical authorization for this research was obtained from the Ministry of Health for the participation of humans (volunteers and collectors) in the experimental hut studies.

### Data analysis

The results from the bioassays were analysed by taking into account the different materials, the presence or absence of a binding resin, and the type and number of washes using a logistic regression model with GLIM 4 software [30]. The effectiveness of the materials was expressed in terms of the probability of survival of the mosquitoes after exposure to the different treated materials. Analysis of variance (ANOVA) of the probability of survival of the mosquitoes as a function of the different variables (material, dose, resin, and type of wash) was performed using the same software.

Concerning the experimental hut evaluation, the effect of each treatment was expressed relative to the control in terms of the proportions of blood feeding and mortality. The proportion of blood feeding was defined as the proportion of blood-fed mosquitoes at the time of collection. Mortality was reported as the proportion of mosquitoes found dead the morning of the collection (immediate mortality) and after 24 hours (delayed mortality).

Proportional data (blood feeding and mortality) obtained from the experimental hut trials were analysed using logistic regression (STATA 6 software, Stata Corporation, College Station, TX, USA).

## Results

### Efficacy and wash resistance of the materials treated with bendiocarb

In total, 350 bioassays were performed using approximately 21,000 *An. gambiae *"Kisumu" females.

**Figure 2:**
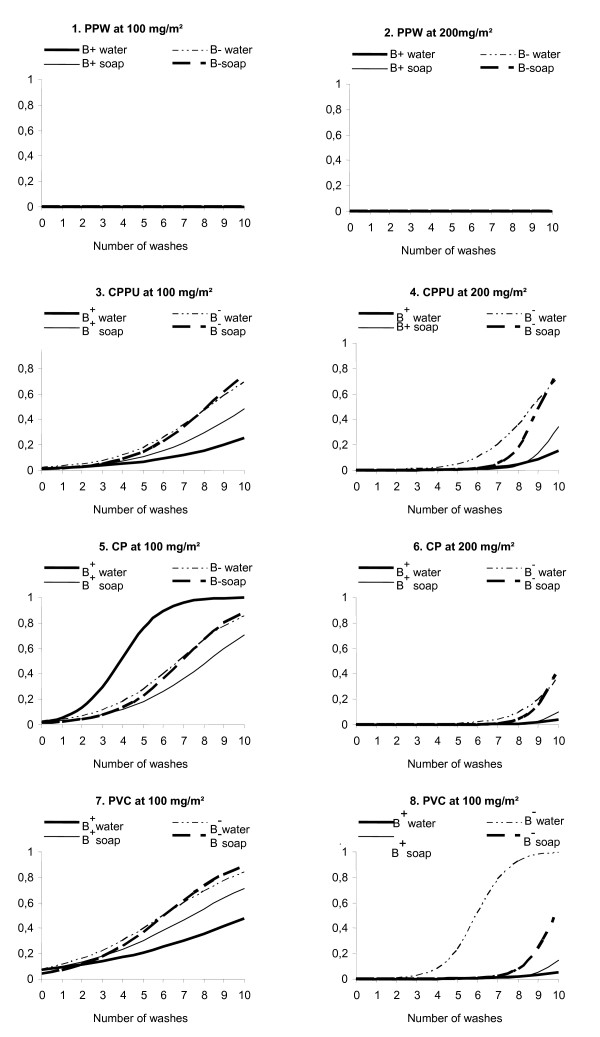
**Probability of survival of *An. gambiae *"Kisumu" exposed to the materials impregnated with bendiocarb as a function of the dose (100 mg/m^2 ^vs. 200 mg/m^2^), the type of liquid used for washing (water vs. soap) and the number of washes (0 to 10)**. Note: B+ = impregnation with binding resin; B- = impregnation without binding resin; water = wash with water alone; soap = wash with soap; P = probability of survival.

Regardless of the dose of bendiocarb used (100 mg/m^2 ^or 200 mg/m^2^), the presence or absence of the binding resin, or the washing method, PPW induced 0% survival (100% mortality) with up to 10 washes (Figure 2). This material, therefore, provided the best results in terms of wash resistance.

The probability of survival after exposure to the other materials depended on the presence or absence of the binding resin and the type and number of washes (Figure 2).

ANOVA showed that all variables (i.e., dose, binding resin, type of wash, and type of material) had significant impacts (p < 0.01) on the probability of survival of the mosquitoes. At a dose of 100 mg/m^2^, the probability of survival of mosquitoes exposed to treated materials was affected by the number of washes more so than at the dose of 200 mg/m^2 ^(p < 0.01). Soap significantly reduced the wash resistance of treated materials (p < 0.01), and the treatment of the materials with binding resin improved the wash resistance (p = 0.025).

### Experimental hut trials

Taking into account the results from the bioassays, we only tested the PPW with fixing resin treated at 200 mg/m^2 ^in the experimental huts. The results are presented in Tables 1 and 2.

**Table 1:** Mortality and blood feeding rates of *Anopheles gambiae *"Ladji" release-recapture in experimental huts (3 replicates)

***An. gambiae***	**Control**	**ITN**	**ITN + PPWI 1/3**	**ITN + PPWI total**
Females caught	206	227	193	202
Females dead	6	92	155	202
Mortality (%)	2.9^a^ [0.6-5.2]	40.5^b^ [34.1-46.9]	80.3^c^ [74.7-85.9]	100^d^
Corrected mortality (%)		38.9^a^	79.7^b^	100^c^
Females blood fed	87	6	15	0
Blood feeding rate (%)	42.2^a^ [35.5-49.0]	2.6^b^ [0.6-4.7]	7.8^c^ [4.0-11.6]	0^b^
Blood feeding inhibition (%)	-	93.74	81.60	100

Note: ITN = insecticide-treated net (deltamethrin at 25 mg/m^2^); PPWI 1/3 = top third of the walls covered with woven polypropylene treated with bendiocarb at 200 mg/m^2^; PPWI total = walls completely covered with woven polypropylene treated with bendiocarb at 200 mg/m^2^. Values in the same row sharing the same superscript letter do not differ significantly (p > 0.05).

**Table 2:** Mortality and blood feeding rates of *Culex quinquefasciatus *"Ladji" after release-recapture in experimental huts (3 replicates)

***Cx. quinquefasciatus***	**Control**	**ITN**	**ITN + PPWI 1/3**	**INT + PPWI total**
Females caught	342	330	286	257
Females dead	2	48	77	121
Mortality (%)	0.6^a^ [0-1.4]	14.6^b^ [10.7-18.4]	26.9^c^ [21.8-32.1]	47.1^d^ [41.0-53.2]
Corrected mortality (%)		14.0^a^	26.4^b^	46.6^c^
Females blood fed	149	24	18	0
Blood feeding rate (%)	43.6^a^ [38.3-48.8]	7.3^b^ [4.5-10.1]	6.29^b^ [3.5-9.1]	0^c^
Blood feeding inhibition (%)	-	83.31	85.55	100

Note: ITN = insecticide-treated net (deltamethrin at 25 mg/m^2^); PPWI 1/3 = top third of the walls covered with woven polypropylene treated with bendiocarb at 200 mg/m^2^; PPWI total = walls completely covered with woven polypropylene treated with bendiocarb at 200 mg/m^2^. Values in the same row sharing the same superscript letter do not differ significantly (p > 0.05).

The mortality rate of *An. gambiae *recorded in the control hut was less than 3%, indicating a lack of insecticide contamination in these huts. With the deltamethrin-treated nets alone, the mortality was 40.5%, increasing to 80.3% and 100% in association with bendiocarb-treated wall coverings on the upper third and complete inside surface of the hut, respectively. The addition of the bendiocarb-treated plastic sheeting to the treated net provided a significant increase in mortality over the net alone (p < 0.001). This increase in mortality was more significant with full coverage of the hut walls (p < 0.001).

The blood feeding rate of *An. gambiae *was 42.2% in the control hut. These rates were considerably less (< 8%) in all other treatments (p < 0.001). It is noteworthy that the addition of the bendiocarb-treated wall covering did not induce any blood feeding inhibition over the treated net alone.

In the control hut, the mortality recorded for *Cx. quinquefasciatus *was only 0.6%. The mortality rates were significantly greater for the other treatments (p < 0.001), with a mortality rate of 14.6% for the treated net alone, 26.9% for the net and upper third wall covering, and 47.1% for the net and full coverage with bendiocarb-treated plastic sheeting. The blood feeding rate in the control hut was 43.5%. This rate was significantly less (< 8%) for all other treatments (p < 0.001).

## Discussion

The present study has evaluated a new vector control technique consisting of the combination of a pyrethroid-treated mosquito net and bendiocarb-treated plastic sheeting to improve the control of pyrethroid-resistant mosquitoes. Results in the laboratory show good wash resistance of woven polypropylene (PPW) compared to other materials, which may be explained by the structure of this material. Some of the insecticide may be retained between the fibres and potentially protected over time from the abrasive effect of the washes. It is also possible that there was an interaction between bendiocarb and polypropylene. The use of soap significantly reduced the wash resistance of treated materials because the half-life of bendiocarb decreases in alkaline solution, and the pH of the soapy water was 9.7. The use of binding resin improved the wash resistance of the treated materials. The resin may form a thin layer, protecting the insecticide against the effects of washing. This resin was similar to that used for bed net impregnation with K-O Tab^® ^1-2-3 (Bayer Environmental Science, Lyon, France) to improve the residual effect of insecticide on the nets.

For the experimental hut study, the results show a weak killing effect of deltamethrin-treated mosquito nets on pyrethroid-resistant *An. gambiae *and *Cx. quinquefasciatus *populations. These results confirmed those of Corbel *et al *[31], who, using experimental huts in Ladji (Benin), showed a low (50%) mortality rate for *An. gambiae *in huts with mosquito nets treated with permethrin at 1 g/m^2^. A more recent study in experimental huts on the same population of *An. gambiae *showed reduced efficacy of nets treated with lambdacyhalothrin at 18 mg/m^2 ^(mortality < 30%), emphasizing the necessity of finding new vector control strategies for malaria control [19].

In this study, the combination of a deltamethrin-treated bed net and a bendiocarb-treated wall covering (with full coverage) was most effective against resistant *An. gambiae*, inducing 100% mortality and 0% blood feeding. Just after releasing mosquitoes in the huts, we noticed that mosquitoes primarily rested on the treated walls, indicating little irritation, perhaps also an effect of the excito-repellent properties of deltamethrin. At this point, the quantity of bendiocarb absorbed through their tarsi may have been sufficient to result in their death or, at least, to change their host-seeking behaviour. The dose of bendiocarb picked up by mosquitoes may also act in synergy with deltamethrin when the mosquitoes leave the wall and come in contact with the net to attempt to feed. Synergism between pyrethroids and carbamates has been documented [32]. Toxicological and electrophysiological studies of synergy between carbamate and pyrethroid insecticides were carried out on two susceptible pest species. The authors proposed a cascade of molecular events to explain the occurrence of synergistic effects between these insecticides [32].

Against *Cx. quinquefasciatus*, this combination was less effective, probably due to the robustness of *Cx. quinquefasciatus *compared to *An. gambiae*. It is likely also due to the high level of insecticide resistance in this species. Experimental hut trials with permethrin-treated nets (Olyset Nets^®^), carried out in an area where pyrethroid resistance is common in Cote d'Ivoire, have shown lower mortality of *Cx. quinquefasciatus *(17%) compared to *An. gambiae *(27.5%) [33]. Another study carried out in southern Benin has shown that carbamate resistance was present at a higher frequency in *Cx. quinquefasciatus *than in *An. gambiae *[10].

Permethrin-treated plastic sheeting was tested against pyrethroid-resistant *An. gambiae *in Burkina Faso [34], showing an efficacy proportional to the surface of the walls covered. However, the mortality rate induced by these treatments was relatively low (44.5% mortality when all four walls were covered), perhaps because of the high frequency of the kdr mutation in the local populations of *An. gambiae *[6] and the high irritability induced in mosquitoes by permethrin [34].

The use of carbamates or organophosphates to treat materials against pyrethroid-resistant *An. gambiae *has already been the subject of several studies [20,21,35-37]. These insecticides have been shown to be effective and constitute good alternatives for control of pyrethroid-resistant malarial vectors, even though they do not confer the same amount of personal protection, due to a weak irritant effect [20]. Their use as a complementary vector control tool in combination with treated nets seems promising.

In the previous studies, carbamates were used on mosquito nets. This may cause concern for human safety, especially that of children because the risk of long periods of contact is greater in this case. In the present study, the coverage of only the upper third of walls with carbamate-treated plastic sheeting, to avoid the risk of contact by children, was tested. The results obtained were satisfactory. The formulation of insecticide used acts by contact. It seems obvious that contact between the user of insecticide treated bed net is more important than contact in the case of insecticide treated plastic sheeting (ITPS) placed at the top of the walls. Although the amount of insecticide used in the case of the ITPS is higher, the direct contact of the user with insecticide appears more reduced. Moreover, it would be possible to develop long-lasting insecticide-treated plastic sheeting (LLIPS). The use of LLIPS as an alternative to IRS has the potential to be widely implemented at the community level, even with relatively limited technical or logistical infrastructure in place.

The present study indicates that the combination of carbamate-treated plastic sheeting and pyrethroid-treated nets is a potential alternative strategy for controlling pyrethroid-resistant vectors, particularly in rural Africa.

## Conclusion

At the moment, African countries and international donors (UNICEF, IMF, WHO, etc.) are investing in the massive distribution of pyrethroid-treated nets to protect populations from malaria. It is vital to study alternative possibilities that will allow the maintenance of the efficacy of this tool and manage insecticide resistance. The mixture of two insecticides with different modes of action has been shown to be a promising strategy for control of pyrethroid-resistant mosquitoes, as well. Pyrethroid resistance constitutes a threat to vector control, as shown by the reduction of efficacy of treated nets [19,31].

These preliminary results should be further studied, including evaluation of entomological, parasitological, and clinical parameters. Selective pressure on resistance mechanisms within the vector population, effects on other pest insects and the acceptability of any control strategy in the community also need to be evaluated.

It is also necessary to continue to look for other methods of vector control. In the short term, the use of new insecticides [38] or even other types of chemicals, alone or in combination, can be effective. One good example is the combination of repellents and insecticides, which has been shown to be promising in areas where *Anopheles *mosquitoes are resistant to pyrethroids [39,40].

## Competing interests

The authors declare that they have no competing interests.

## Authors' contributions

AD and FC conceived of the study. AD and FC have participated in the design of the study. AD, FC, and CP have participated in the analysis and interpretation of data. AD carried out bioassays in laboratory. AD and JC carried out data collection in experimental huts. The manuscript has been drafted by AD. AD, FC, TB, SI, J-M H, VC, and MA have been involved in manuscript revising. All authors read and approved the final manuscript.
